# Challenges in Diagnosis and Management of Ovarian Neuroendocrine Carcinoma: A Case of Aggressive Disease With Multimodal Treatment Approach

**DOI:** 10.1002/ccr3.71392

**Published:** 2025-10-30

**Authors:** Javeria Haider, Humera Mahmood, Muhammad Faheem, Shaista Khurshid, Abdullah, Biruk Demisse Ayalew, Humza Saeed

**Affiliations:** ^1^ Department of Oncology Atomic Energy Cancer Hospital NORI Islamabad Pakistan; ^2^ Department of Internal Medicine Rawalpindi Medical University Rawalpindi Punjab Pakistan; ^3^ St. Paul's Hospital Millennium Medical College Addis Ababa Ethiopia

**Keywords:** carboplatin, chemotherapy, cisplatin, etoposide, neuroendocrine tumors, ovarian, paclitaxel

## Abstract

Neuroendocrine tumors (NETs) of the ovary are extremely rare, accounting for only 1%–2% of malignant ovarian tumors, with high‐grade subtypes demonstrating particularly aggressive behavior. We describe a 47‐year‐old woman who presented with abdominal swelling, irregular bleeding, and pain, and was diagnosed with high‐grade ovarian neuroendocrine carcinoma following surgical resection. Despite initial treatment with paclitaxel and carboplatin, residual disease persisted, prompting therapy with long‐acting Sandostatin. The disease subsequently progressed with hepatic and peritoneal involvement, and she was successfully managed with cisplatin and etoposide. This case illustrates the diagnostic complexity and aggressive course of ovarian neuroendocrine carcinoma and underscores the importance of individualized, multimodal treatment strategies and vigilant follow‐up in the absence of standardized guidelines.

**Abbreviations**
ACT: atypical carcinoid
LCNEC: large‐cell neuroendocrine carcinoma
NENs: neuroendocrine neoplasms
NETs: Neuroendocrine tumors
NSCNEC: non‐small‐cell neuroendocrine carcinoma

**Summary**

- Ovarian neuroendocrine carcinomas are exceptionally rare and aggressive tumors with no standardized treatment guidelines, often leading to diagnostic and therapeutic challenges.
- This case emphasizes the need for individualized multimodal management, including surgery, chemotherapy, and somatostatin analogues guided by tumor biology.
- Continuous monitoring and timely therapeutic adjustments are essential to improve outcomes in these patients.

## 1 Introduction

Neuroendocrine tumors (NETs) are aggressive diseases that most often arise in the gastrointestinal tract, pancreas, and lungs. Cases occurring in other tissues and organs are rare, especially in the female reproductive tract [1].

Ovarian NETs account for only 1%–2% of malignant ovarian tumors. Broadly, these tumors are divided into carcinoid, atypical carcinoid (ACT), small‐cell carcinoma, and large‐cell neuroendocrine carcinoma (LCNEC) types. According to World Health Organization (WHO) regulations, non‐small‐cell neuroendocrine carcinoma (NSCNEC) is similar to LCNEC. Carcinoids and ACT are classified as low‐grade NETs, whereas small‐cell carcinoma, LCNEC, and NSCNEC are classified as high‐grade neuroendocrine carcinomas. High‐grade neuroendocrine carcinomas are considered more aggressive than low‐grade NETs [2].

Due to the low incidence of ovarian NETs, there are no standard treatment guidelines for the disease and factors influencing patient prognosis. This can also result in missed diagnosis or treatment delay in such cases [3].

This case report aims to illustrate the complexities involved in diagnosing and managing ovarian NETs, which are rare and aggressive malignancies with no standard treatment guidelines. By presenting this case, we seek to highlight the need for individualized treatment approaches and increase awareness among clinicians to improve patient outcomes and reduce the risk of missed diagnoses or treatment delays.

## 2 Case Presentation

**FIGURE 1:**
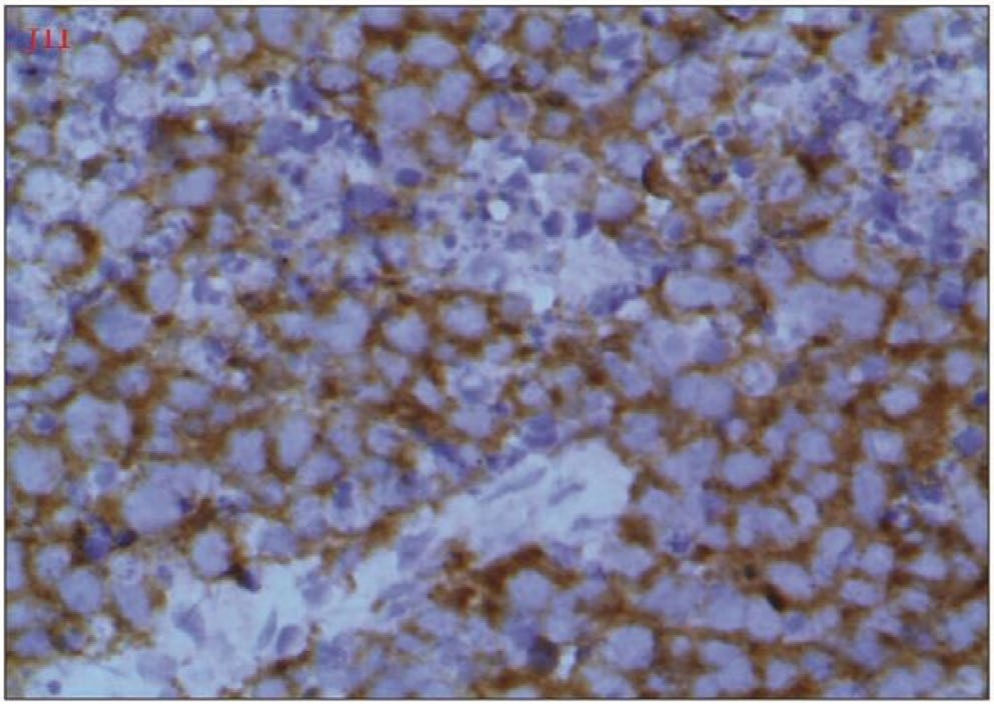
Immunohistochemistry (IHC) results showing PAX‐8 positivity, WTI negativity, ER positivity, and synaptophysin positivity, indicating neuroendocrine differentiation. The Ki‐67 index was high, reflecting tumor aggressiveness, while calretinin and CD99 were negative.

**FIGURE 2:**
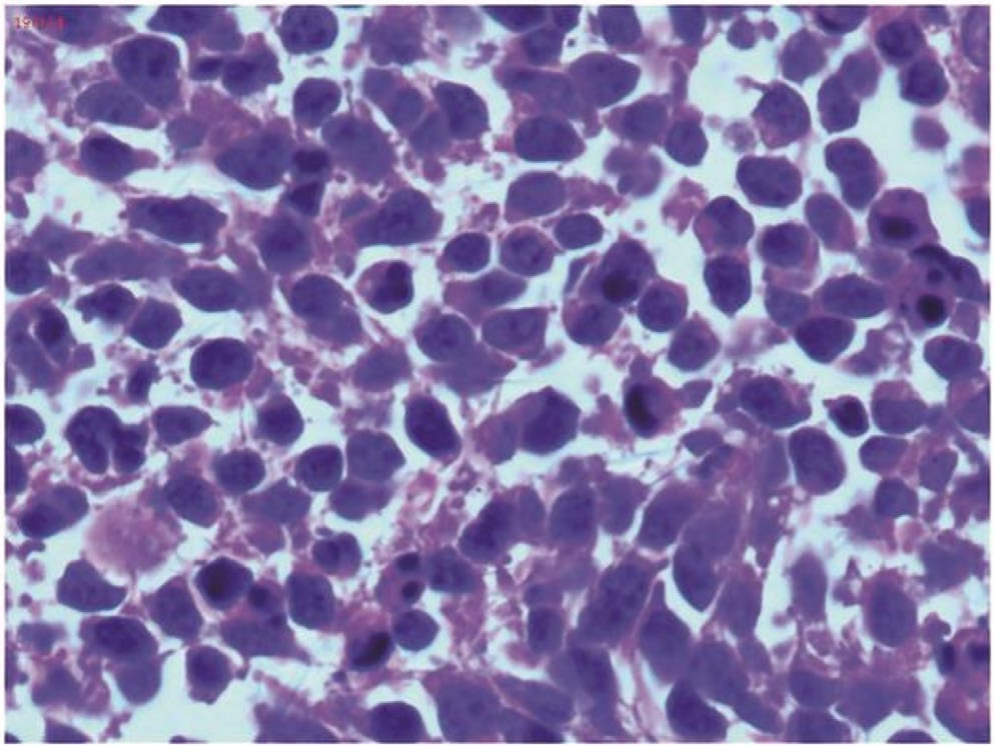
H&E stain of neuroendocrine tumor. The tissue section shows hypercellularity and the majority of the tumor cells is small and hyperchromatic with high nuclear cytoplasmic ratio.

We report the case of a 47‐year‐old female, in her usual state of health with no significant past medical or surgical history. There is no history of any known drug allergies or smoking or drinking alcohol. She is a housewife and is para 4 and all were normal vaginal deliveries. It was until 4 months back when she presented with complaints of abdominal pain, distension, and irregular menstrual bleeding. Initially she was evaluated at a local hospital with only an ultrasound scan on which she was found to have a thick‐walled cystic mass just above the uterine fundus, occupying most of the pelvic cavity, and bilateral ovaries could not be visualized. She underwent total abdominal hysterectomy with bilateral salpingo‐ophorectomy (TAH + BSO) at a local hospital. Histopathology of the surgical specimen confirmed the diagnosis of neuroendocrine carcinoma. Immunohistochemistry (IHC) showed PAX‐8 positive, WTI negative, ER positive, synaptophysin positive, with a high Ki‐67 index, while calretinin and CD99 were negative (Figure 1). Histopathological examination with H&E staining revealed hypercellularity, with the majority of tumor cells appearing small and hyperchromatic, showing a high nuclear‐cytoplasmic ratio (Figure 2). The above‐mentioned markers were applied keeping in view the suspicion of ovarian carcinoma but synaptophysin positivity and the presence of small, hyperchromatic cells suggested neuroendocrine histology, but high Ki‐67 shows a high‐grade tumor.

## 3 Differential Diagnosis, Investigations, and Management

**FIGURE 3:**
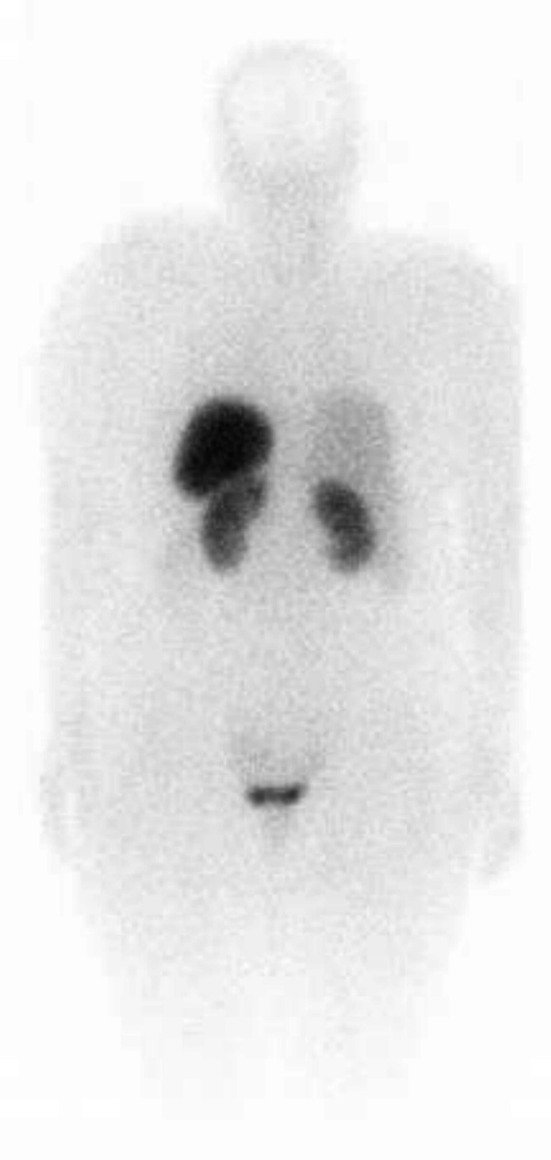
Post‐operative/pre‐chemotherapy octreotide scan.

**FIGURE 4:**
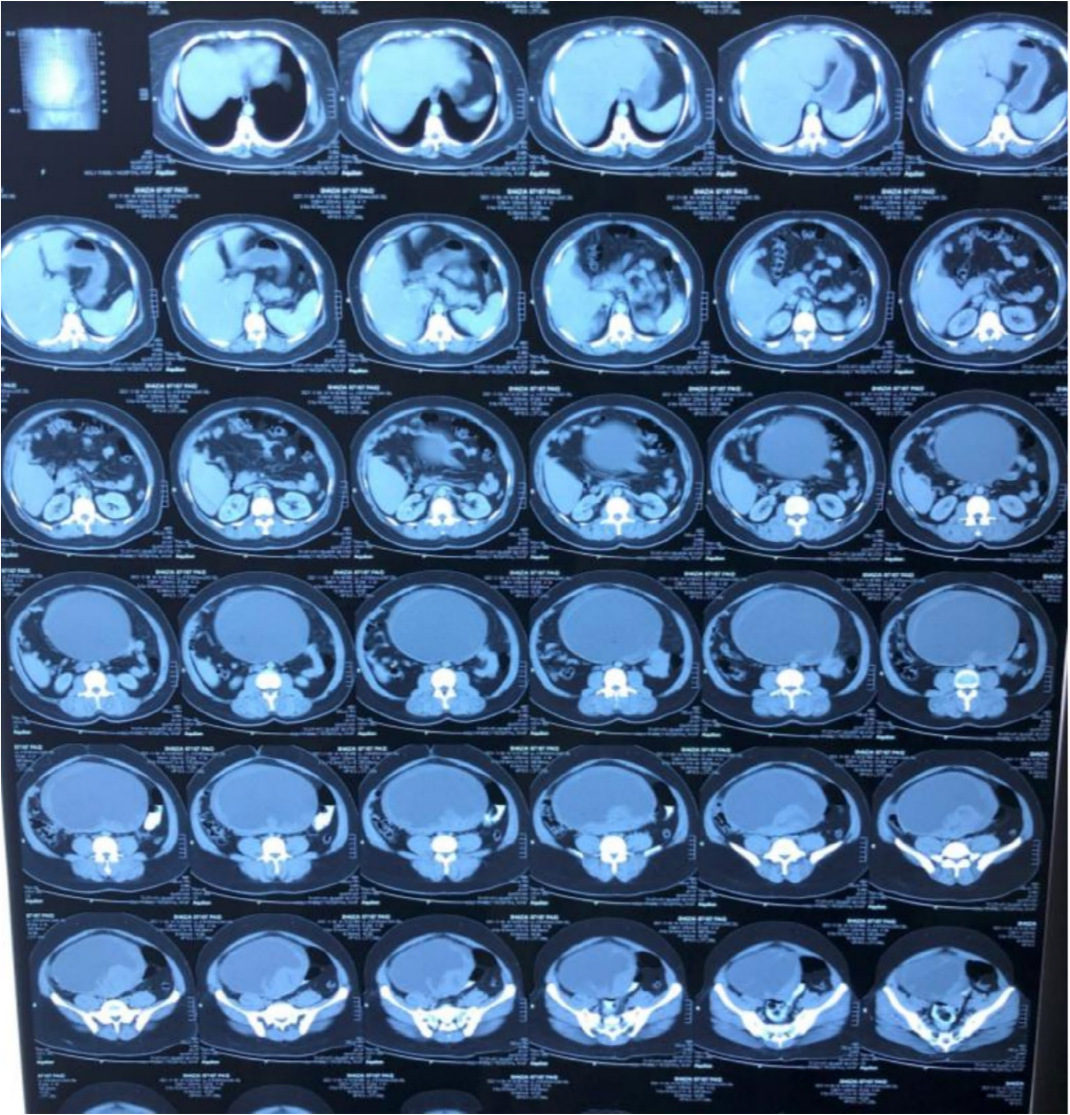
Post‐operative/pre‐chemotherapy CT scan.

**FIGURE 5:**
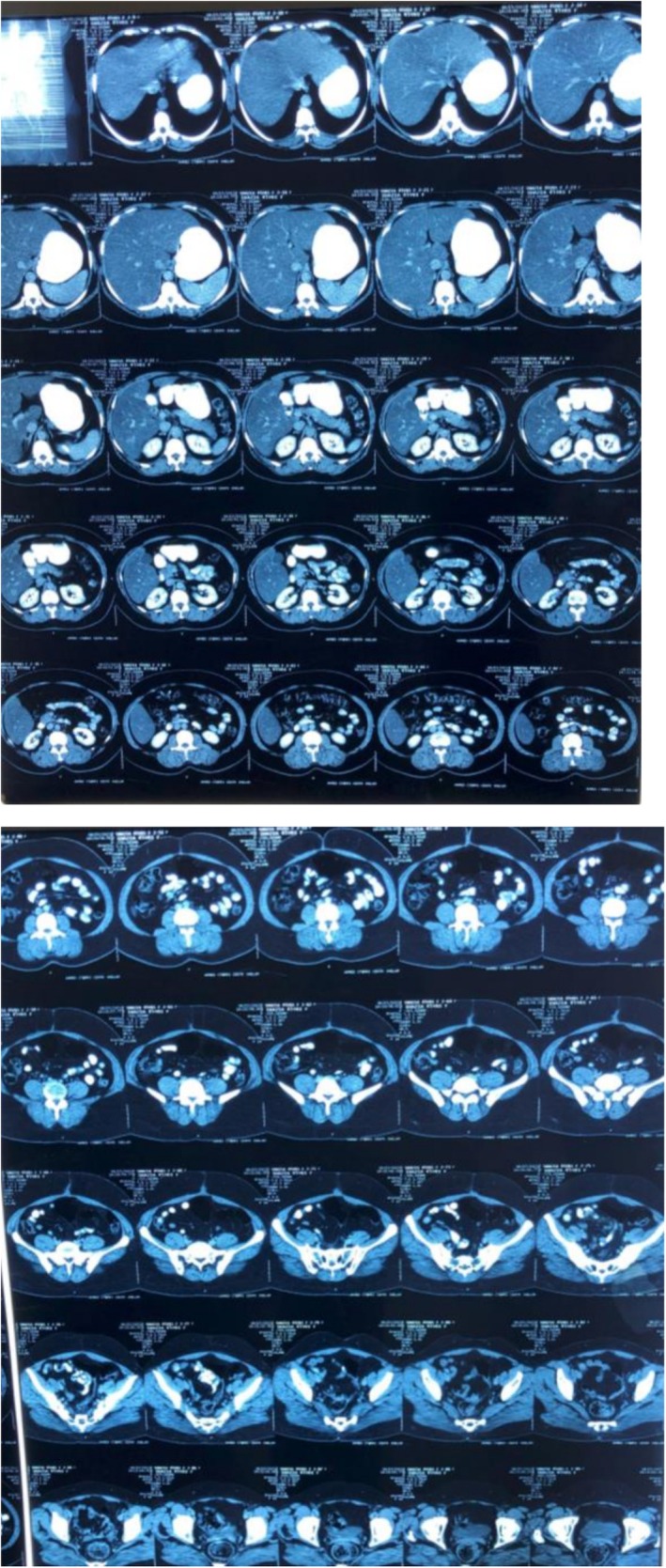
CT scan post four cycles of paclitaxel/carboplatin CT scan.

Post‐operatively, the patient experienced persistent abdominal distension and pain in the right hypochondrium. An octreotide scan was advised due to the suspicion of residual NET, as most neuroendocrine tumors show sandostatin receptor uptake, which showed mild increased radiotracer uptake involving the vaginal stump and the left side of the pelvis, indicating possible post‐surgical sequelae or residual disease (Figure 3). A post‐operative CT scan also revealed hypodense lesions in the liver parenchyma (Figure 4), but these did not show uptake on the octreotide scan. In our patient the tumor cells showed high proliferative potential so that could be one of the reasons for this mixed picture (some part of residual disease showed octreotide activity while others did not). Ideally all patients that are suspicious of having malignancy should undergo staging workups prior to any intervention, but in our case our patient was operated up front at a local hospital, so we only had to rely on post‐operative disease status. The patient was initially started on chemotherapy with paclitaxel and carboplatin as part of the residual disease on CT showed no activity on the octreotide scan and the tumor cells showed a high proliferative index. She was planned on a three weekly chemotherapy schedule consisting of 175 mg/m^2^ of intravenous (IV) paclitaxel and three weekly IV carboplatin using area under curve (AUC) 5 of which she received four cycles with a good subjective response; and the patient did not experience any major side effects like arthralgia, myalgias or neuropathy. However, residual disease remained on the follow‐up CT scan (Figure 5). Upon reviewing the case and considering the mild radiotracer uptake on the octreotide scan and the stable hepatic lesions on interval imaging, the patient was switched to LAR Sandostatin, of which she received six cycles. Sandostatin is considered a first‐line treatment option in most low‐grade neuroendocrine tumors because of its antagonist activity against sandostatin receptors and is generally well‐tolerated. A follow‐up octreotide scan showed no avid lesions, and the patient was placed on routine follow‐up.

## 4 Outcome and Follow‐Up

**FIGURE 6:**
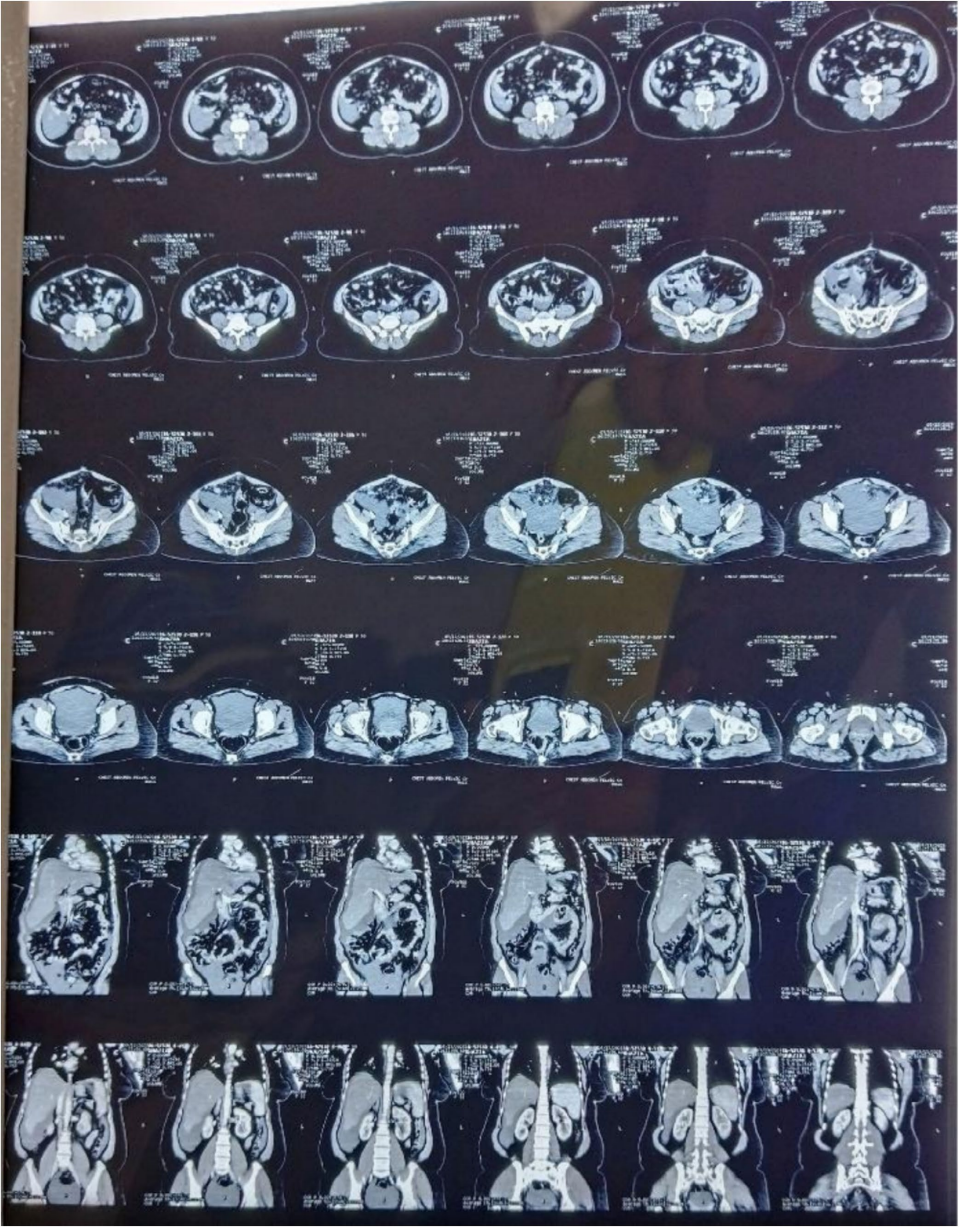
Follow‐up CT scan before carboplatin/etoposide.

Six months later, during a follow‐up visit, the patient presented with recurrent abdominal pain, more pronounced in the right hypochondrium. A repeat CT scan showed disease progression involving the liver and the appearance of omento‐peritoneal deposits (Figure 6), which were suspicious for disease recurrence. Due to limited resources and the patient's unwillingness, no new biopsy was performed at that time. Given the initial response to LAR Sandostatin, the patient was rechallenged with this treatment, but symptoms did not improve. Consequently, she was switched to a second‐line chemotherapy regimen with cisplatin and etoposide using a three weekly protocol with 75 mg/m^2^ IV cisplatin and 100 mg/m^2^ IV etoposide, receiving five cycles with a favorable clinical and radiological response. The patient was able to tolerate chemotherapy well and experienced only mild neuropathy. She is now on regular follow‐up as there is a good response but considering the high risk of recurrence considering the ovarian primary she will be kept on surveillance with ultrasound and CT scan. There are no necessary lifestyle modifications or dietary restrictions that can help prevent recurrence as she was already leading a balanced, healthy life. However, close follow‐up and monitoring are necessary for early detection of disease so that treatment can be initiated before the development of worsening symptoms.

Several alternative options like Peptide receptor radionuclide directed therapy (PRRT) and novel targeted therapy targeting tyrosine kinase and mTOR pathway are available but unfortunately because of limited resources and their cost they could not be used in our patient. Moreover, since the tumor was high grade and the symptomatic metastatic disease burden was high, she was started on chemotherapy instead.

## 5 Discussion

NETs are aggressive neoplasms that most commonly occur in the gastrointestinal tract, pancreas, and lungs. Occurrences in other tissues, particularly the female reproductive tract, are rare [4]. Ovarian NETs account for only 1%–2% of malignant ovarian tumors. These tumors are generally divided into carcinoid, ACT, small‐cell carcinoma, and LCNEC types. LCNEC and NSCNEC are classified as high‐grade neuroendocrine carcinomas due to their aggressive nature [5].

According to the World Health Organization's 2020 classification of gynecologic neuroendocrine neoplasms (NENs), there are only two categories: well‐differentiated tumors (NETs) and poorly differentiated carcinomas (NECs). The well‐differentiated neuroendocrine tumors follow a prolonged clinically indolent course whereas poorly differentiated tumors have a high proliferative index and behave like aggressive carcinomas [1]. For ovarian neuroendocrine tumors, the WHO still recommends using the term “ovarian carcinoid” instead of “well‐differentiated neuroendocrine tumor” [6]. Most ovarian NENs are associated with epithelial ovarian cancer or ovarian teratomas, displaying focal neuroendocrine differentiation [7]. Pure ovarian NENs are extremely rare, with only a few cases reported in the literature [8]. In our case there was no evidence of epithelial ovarian cancer or features suggestive of teratoma so it likely seemed as primary ovarian neuroendocrine cancer.

Ovarian NETs are often detected incidentally, although some patients may present with nonspecific symptoms such as abdominal discomfort, persistent facial flushing, episodic hypertension, bloating, nausea, vomiting, and abdominal distension due to ascites [9]. Similarly in our patient there were nonspecific symptoms like abdominal pain and distension which can be misleading and lead to diagnostic difficulties.

The primary pathological tool for diagnosing neuroendocrine neoplasms is immunohistochemistry. For diagnosing a neuroendocrine neoplasm of the female gynecological tract, at least two positive neuroendocrine markers, such as synaptophysin, chromogranin, and CD56, are required. The IHC Panel applied to our patient's histopathology sample also showed synaptophysin positivity and the microscopy also showed features suggestive of neuroendocrine cancer. The protein Ki‐67 is a marker for tumor aggressiveness and is closely associated with cell proliferation [10]. This marker helps guide treatment decisions and determines the use of diagnostic tools, as tumors with a Ki‐67 index of less than 30% are more likely to be detected using somatostatin receptor imaging [8]. The tumor cells in our case also showed a high proliferative index (high Ki‐67), and that could be one of the reasons for the mixed radiotracer uptake in the octreotide scan.

Due to the low incidence of ovarian NETs, there are no standard treatment guidelines, leading to potential missed diagnoses or treatment delays. Most cases are reported in small series, making comprehensive studies difficult. Ovarian NETs are typically treated according to ovarian cancer guidelines; however, despite their usual early‐stage diagnosis, these tumors can be more aggressive. Low‐grade NETs usually present at stage I and have lower rates of lymph node involvement and distant metastasis than high‐grade neuroendocrine carcinomas. Since our patient had high‐grade neuroendocrine disease, there was metastatic involvement of the liver. Surgery remains the primary treatment, with total abdominal hysterectomy and bilateral salpingo‐oophorectomy (TAH + BSO) preferred for complete macroscopic removal, except in young patients requiring fertility preservation [3, 11].

Platinum‐based chemotherapy, such as cisplatin or carboplatin with etoposide, is preferred in the adjuvant setting for high‐grade NETs [3]. For low‐grade NETs, somatostatin analogues or PRRT may be considered, provided a positive somatostatin receptor scan is obtained before therapy. The efficacy of these options appears limited to well‐differentiated neoplasms and should only be considered for poorly differentiated tumors when the Ki‐67 index is below 30% [8]. In our patient, due to mild uptake on the octreotide scan, she was treated with both lines of chemotherapy. But in the case of advanced, resistant and recurrent settings PRRT can be offered as a means of treatment as well.

## 6 Conclusion

This case report highlights the challenges in diagnosing and treating rare and aggressive ovarian neuroendocrine tumors (NETs), for which no standardized treatment guidelines exist. These patients normally present with vague symptoms like abdominal pain and distension which can delay the diagnosis. The patient's management involved a combination of surgery, chemotherapy, and somatostatin analogues, tailored to the tumor's specific characteristics. The case emphasizes the importance of personalized treatment strategies, continuous monitoring, and collaboration among clinicians to improve outcomes for patients with ovarian NETs. It also points out how a tumor can present differently and can show different radiotracer activity. In our patient, the disease involving the liver parenchyma showed no octreotide activity and thus could not be controlled with Sandostatin alone, so chemotherapy had to be added.

## Author Contributions

**Javeria Haider:** conceptualization, data curation, supervision, writing – original draft, writing – review and editing. **Humera Mahmood:** data curation, resources, supervision, validation, writing – original draft, writing – review and editing. **Muhammad Faheem:** conceptualization, supervision, validation, visualization, writing – original draft, writing – review and editing. **Shaista Khurshid:** data curation, investigation, writing – original draft, writing – review and editing. **Abdullah:** data curation, investigation, methodology, writing – original draft, writing – review and editing. **Biruk Demisse Ayalew:** conceptualization, supervision, validation, writing – original draft, writing – review and editing. **Humza Saeed:** conceptualization, investigation, project administration, writing – original draft, writing – review and editing.

## Consent

Written informed consent was obtained from the patient to publish this report per the journal's patient consent policy.

## Conflicts of Interest

The authors declare no conflicts of interest.

## Data Availability

Data and materials are available upon request from the corresponding author.
